# Artificial intelligence simulation study of Therapeutic Plasma Exchange (TPE) using an 8- State Markov State-Transition Model integrated within a Bayesian Markov Chain Monte Carlo Framework

**DOI:** 10.21203/rs.3.rs-11283568/v1

**Published:** 2026-10-21

**Authors:** Vladimir Kuklin, Philip Keith, Timofey Kondratiev, L. Keith Scott, Bernd Stegmayr, Sascha David, Joseph A. Carcillo

## Abstract

Introduction Sepsis-induced organ dysfunction remains a leading cause of mortality in critical care. While recent evidence suggests that Therapeutic Plasma Exchange (TPE) may improve outcomes, significant heterogeneity among septic patient populations in prior trials might have contributed to mixed responses and thus fueled clinical skepticism. To address this challenge and to model TPE effectiveness across varying levels of sepsis severity, we utilized a time-dependent, 8-state transition Markov model integrated with a Bayesian Markov Chain Monte Carlo (MCMC) framework. Methods An AI-generated "digital twin" cohort (N = 10,000) was randomized 1:1 to standard care (SC) or adjuvant TPE groups. Transition probabilities were parameterized using a pooled relative risk (RR) of 0.58, derived from five recent meta-analyses. The model incorporated specific hazard multipliers for acute kidney injury (AKI; 1.5x), thrombocytopenia (1.3x), and high-dose vasopressor requirements (1.25x). Patient flow was simulated through eight clinically defined health states over a 28-day horizon. Outcomes included 28-day survival, absolute risk reduction (ARR), and number needed to treat (NNT) stratified by Sequential Organ Failure Assessment (SOFA) score quartiles (8, 12, 16, and 20). Bayesian methods were employed to determine survival probabilities and hazard ratios (HR) with 95% credible intervals (CrI). Results Across the modelled full 10,000-patient cohort, TPE increased 28 day survival compared to standard care (67.3% vs. 44.7%). The survival benefit was modest in the group with the lowest degree of organ failure (SOFA 8) (TPE 87.6% vs. SC 78.4%; ARR 9.2%; NNT 11). However, this benefit was more pronounced in cases of higher burden of organ failure, particularly within the SOFA 20 cohort (TPE 48.8% vs. SC 12.8%; ARR 36%; NNT 3) and in cases with AKI + thrombocytopenia + higher doses of vasopressors (ARR 36%; NNT 3). For SOFA 20 patients, TPE was associated with a 38% reduction in the hazard of death (HR 0.62; 95% CrI 0.54–0.71). Conclusion These simulation results suggests that the efficacy of TPE might be dependent on baseline illness severity. While the clinical impact is more limited in moderate sepsis (SOFA 8), TPE appears to offer a profound survival advantage and high therapeutic yield for patients with multi-organ failure.

